# Long forms of cardiac troponin T for myocardial infarction diagnosis: the SuperTROPO study

**DOI:** 10.1093/eurheartj/ehaf975

**Published:** 2025-12-05

**Authors:** Konsta Teppo, K E Juhani Airaksinen, Tuija Vasankari, Anna Linko-Parvinen, Hanna-Mari Pallari, Tuomas Paana, Samuli Jaakkola, Helea Junes, Selma Salonen, Tuulia Tuominen, Sara Simonen, Marjatta Strandberg, Tapio Hellman, Saara Wittfooth

**Affiliations:** Heart Center, Turku University Hospital, Turku, Finland; Biotechnology Unit, Department of Life Technologies, University of Turku, Turku, Finland; Heart Center, Turku University Hospital, Turku, Finland; Heart Center, Turku University Hospital, Turku, Finland; Tyks Laboratories, Clinical Chemistry, Turku University Hospital, Turku, Finland; Department of Clinical Chemistry, University of Turku, Turku, Finland; Tyks Laboratories, Clinical Chemistry, Turku University Hospital, Turku, Finland; Heart Center, Turku University Hospital, Turku, Finland; Heart Center, Turku University Hospital, Turku, Finland; Biotechnology Unit, Department of Life Technologies, University of Turku, Turku, Finland; Biotechnology Unit, Department of Life Technologies, University of Turku, Turku, Finland; Biotechnology Unit, Department of Life Technologies, University of Turku, Turku, Finland; Biotechnology Unit, Department of Life Technologies, University of Turku, Turku, Finland; Emergency Department, Turku University Hospital, Turku, Finland; Kidney Center, Turku University Hospital, Turku, Finland; Biotechnology Unit, Department of Life Technologies, University of Turku, Turku, Finland

**Keywords:** Myocardial infarction, Type 1 myocardial infarction, Cardiac troponin, Long cTnT, Troponin fragmentation

## Abstract

**Background and Aims:**

Elevated cardiac troponin levels are a frequent finding in emergency department patients, often without a clear cause. Current high-sensitivity cardiac troponin T (cTnT) assays measure intact and fragmented cardiac troponin T (total cTnT) molecules, without distinguishing between them. This study investigated whether measuring only intact and minimally fragmented cTnT (long cTnT) provides additional value for myocardial infarction (MI) identification.

**Methods:**

Consecutive emergency department patients with standard high-sensitivity cTnT levels (Roche Diagnostics) above the upper reference limit (≥14 ng/L) were recruited. Long cTnT levels were measured using a novel immunoassay. The additional diagnostic value of long cTnT in identifying patients with type 1 MI or any MI was assessed.

**Results:**

A total of 1811 patients participated in the study, 1145 (63.2%) presenting with chest pain or dyspnoea. Overall, 205 (11.3%) had MI, including 148 classified as type 1 MI. Only .7% of patients in the lowest long cTnT tertile (<3.7 ng/L) had type 1 MI. The discriminative ability of long cTnT was superior to total cTnT in identifying patients with MI (area under curve [95% confidence intervals]) for any MI: .833 (.804–.863) vs .782 (.744–.819), and for type 1 MI: .839 (.807–.872) vs .777 (.735–.819), both (*P* < .001). Integrating the predictive data from long cTnT with total cTnT provided additional value in both reclassification and decision curve analyses, compared to total cTnT data alone.

**Conclusions:**

The long cTnT assay demonstrated good diagnostic performance in identifying MI in patients with elevated total cTnT levels, with the potential to improve the accuracy of MI diagnosis.

## Structured Graphical Abstract

**Structured Graphical Abstract:**
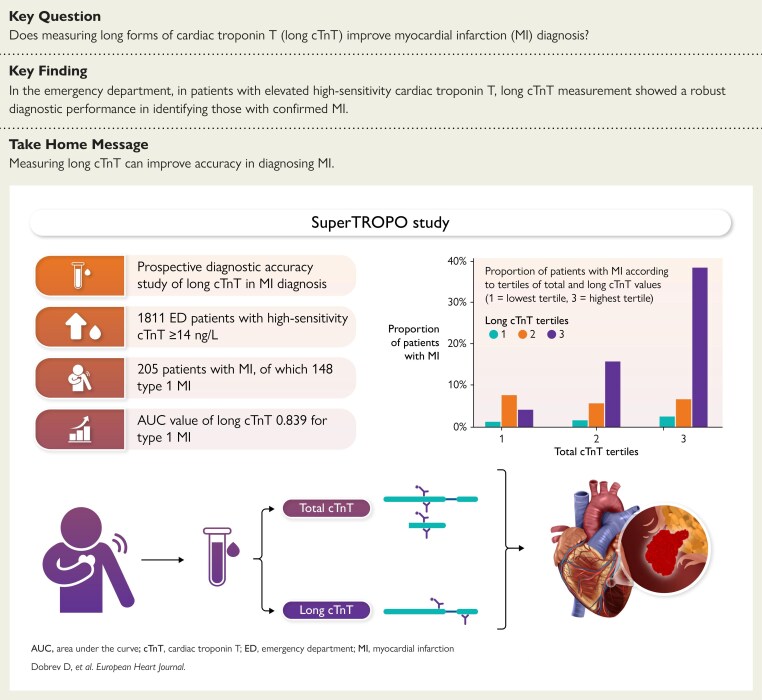
Long forms of cardiac troponin T in the diagnosis of myocardial infarction. AUC, area under the curve; cTnT, cardiac troponin T; ED, emergency department; MI, myocardial infarction.

**See the editorial comment for this article ‘Long form troponins: a promising new diagnostic tool but not quite ready for prime time’, by C. W. Hamm, https://doi.org/10.1093/eurheartj/ehaf803.**

## Introduction

Cardiac troponin measurements play an important role in diagnosing acute coronary syndromes.^1^ The introduction of high-sensitivity cardiac troponin assays has made minor troponin elevations a frequent finding in clinical practice.^2,3^ However, the vast majority of troponin elevations is not caused by acute myocardial infarction (MI), and even fewer by type 1 MI—a condition characterized by acute coronary atherothrombosis, requiring accurate diagnosis and an early invasive treatment strategy.^2,4^ Indeed, several clinical conditions other than MI are also known to cause transient or sustained troponin elevations, complicating the diagnosis of MI, and often leading to unnecessary and potentially harmful antithrombotic therapy and invasive coronary angiography.^5–9^

In MI, cardiac troponins are released from ischaemic myocardial tissue as a combination of full-length molecules and troponin fragments, which then undergo progressive proteolytic degradation.^5,6,10,11^ However, the exact mechanisms of troponin release from the myocardium are not completely understood.^12^ We hypothesized that particularly in type 1 MI, which typically has a more abrupt onset and more profound myocardial damage than type 2 MI, troponin elevations would consist mainly of intact or longer troponin molecules. In myocardial injury, smaller cardiac troponin fragments have been shown to be responsible for troponin elevations, as observed in conditions such as strenuous exercise, Takotsubo syndrome, end-stage kidney disease, and chronic cardiomyopathies.^9,13–18^ The troponin release in these settings might be associated with reversible mechanisms, such as an increase in cell membrane permeability, rather than myocardial cell death.^12^

Importantly, the current commercial high-sensitivity cardiac troponin T (cTnT) assays measure both the longer cTnT molecules and the highly fragmented shorter forms without distinguishing between them. The inability of these ‘total cTnT’ assays to differentiate between molecule lengths may contribute to their limited specificity for MI. We have recently developed a novel immunoassay to measure intact and mildly truncated cTnT forms, referred to as long cTnT, which is both simpler and more sensitive than the time-consuming methods previously used to investigate troponin fragmentation.^9,19^ In this study, we aimed to assess whether measuring long cTnT provides additional value in identifying MI, particularly type 1 MI, amongst emergency department (ED) patients with total cTnT levels above the 99th percentile upper reference limit in real-life clinical practice.

## Methods

### Study cohort

The prospective SuperTROPO study (ClinicalTrials.gov Identifier: NCT05858112) recruited consecutive patients with total cTnT levels above the upper reference limit (≥14 ng/L) at the ED of Turku University Hospital, Turku, Finland, between 8 May 2023, and 30 May 2024 (with a summer pause in recruitment from 22 June 2023, to 1 September 2023). The inclusion criteria were an ED visit, age >18 years, a high-sensitivity total cTnT value ≥14 ng/L upon arrival at the first blood sample, and signed informed consent. Only patients with total cTnT levels above the upper reference limit were included, as there is a clinical need to improve the diagnostic accuracy for MI in patients with elevated troponin levels. Exclusion criteria included inability to provide informed consent, a vulnerable condition (delirium, dementia, or critical illness), pregnancy, and prior participation in the study. The ‘cardiac package’ of laboratory tests, including the high-sensitivity total cTnT measurement, which was used to screen for study inclusion (≥14 ng/L), was ordered at the discretion of the attending clinician. This package is typically ordered in the clinical practice of Turku University Hospital ED when a cardiac event is suspected or when symptoms are unclear, and the possibility of a cardiac event cannot be ruled out. In addition to high-sensitivity total cTnT, it includes complete blood count, plasma C-reactive protein, sodium, potassium, and creatinine.

Patients meeting the inclusion criteria were asked for their consent to participate in the study. Patients were recruited from hospital wards or, if discharged early from the ED, via mail. Twenty-five per cent of the potential study participants were recruited in person during the hospital stay, and only a few of them (22 patients) refused to participate. The consent rate was slightly below 40% in the discharged patients to whom informed consent forms were mailed. In total, 4837 samples were collected. After excluding samples from individuals who did not provide consent, those who had previously participated in the study (only the first ED visit was included), and samples that were insufficient or haemolytic, a total of 1811 patients (38%) were included in the study (see Supplementary data online, *Figure S1*).

The diagnostic value of troponin measurements has traditionally been validated in selected cohorts of patients presenting with potential symptoms of MI, yet defining symptom status in clinical practice is often challenging. Moreover, minor troponin elevations present diagnostic difficulties, particularly in patients without typical symptoms or findings suggestive of MI. Therefore, our primary analysis focused on a broader cohort, i.e. all patients presenting to the ED with elevated cTnT levels, regardless of the type of acute symptoms. Additionally, we assessed the performance of cTnT assays separately in patients presenting with chest pain or dyspnoea.

### Definition of myocardial infarction

The main outcome, type 1 MI, was defined in accordance with current clinical guidelines.^1,20^ In brief, type 1 MI was defined as a clinical setting leading to acute myocardial ischaemia from an acute coronary atherothrombosis. A secondary outcome was any MI, including all cases with evidence of myocardial necrosis and clinical features indicative of ischaemia. Additionally, we evaluated patients with type 2 MI, defined as acute myocardial ischaemia resulting from a mismatch between oxygen supply and demand in the myocardium, unrelated to acute coronary atherothrombosis. Only MI types 1 and 2 were observed in this cohort, and thus, the term ‘any MI’ refers in this study to patients with either type 1 or type 2 MI.

The final MI diagnosis was reviewed from patient records of the index hospitalization and adjudicated by two cardiologists based on all available clinical data: routine laboratory tests (including dynamic changes in total cTnT levels in the ED and during hospitalization), electrocardiogram, echocardiography, and imaging findings, including cardiac computed tomography angiography and conventional angiography. No single algorithm for total cTnT measurement was mandated, as serial cTnT sampling was performed at the discretion of the treating clinicians when deemed necessary, but the national guidelines on acute coronary syndromes advocate the use of the 0/1 h or 0/2 h algorithms when clinically applicable.^21^ A third cardiologist was consulted to resolve any disagreements. All adjudicators were blinded to long cTnT values.

### Blood sampling

The first routine laboratory samples were collected directly at admission to the ED. The samples were analysed fresh for total cTnT as part of the normal clinical practice. Leftover lithium-heparin plasma samples obtained during this routine baseline (0 h) testing were used as study samples. After total cTnT analysis before centrifugation, the leftover samples were kept at +4°C. The centrifuged plasma samples were aliquotted into coded vials and frozen at −70°C within 12 h of sampling. A stability study was conducted to confirm analyte stability at room temperature and at +4°C during the maximum period of 12 h between sampling and sample freezing.

### Total cardiac troponin T assay

All plasma samples were analysed for total cTnT with the Elecsys Troponin T high-sensitivity kit using the Cobas 8000 system (e801 module) (Roche Diagnostics GmbH, Mannheim, Germany). The Elecsys Troponin T high-sensitivity assay uses two monoclonal antibodies (mAb), which specifically target the central part of human cTnT and detect intact, mildly, and heavily fragmented cTnT forms. For this assay, as reported by the assay manufacturer in the package insert, the measuring range is 3–10 000 ng/L with a limit of detection of 3.0 ng/L and a limit of quantitation [coefficient of variation (CV) ≤10%] of 13 ng/L.

### Long cardiac troponin T assay

Our novel highly sensitive two-step heterogeneous sandwich-type immunoassay based on upconversion luminescence was used for the detection of long (intact and mildly fragmented) molecular forms of cTnT.^19^ The assay was performed according to the previously published protocol except the capture antibody and sample incubations were 60 min instead of 30 min.^19^ These modifications were made to streamline the analysis process, and they were experimentally proven not to impact the performance of the assay. All study samples were analysed in duplicates in batch format. The anti-cTnT mAbs and human cardiac troponin ITC complex used as a calibrator were obtained from HyTest Ltd (Turku, Finland). The capture antibody (7E7 mAb) and the tracer antibody (1C11 mAb) bind to amino acid residues (aar) 223–242 and 174–190 of cTnT, respectively. The C-terminal region of cTnT between these two epitopes (aar 189–223) contains several cleavage sites, and thus, the ability of the assay to detect long forms of cTnT is based on targeting all cTnT molecules that are not degraded at aar 189–223. The limit of detection and limit of quantitation (CV 10%) of this assay are .4 and 1.8 ng/L, respectively. The ratio of long cTnT forms to total cTnT (troponin ratio) was used as the measure of troponin fragmentation.

### Study ethics

The study complies with the Declaration of Helsinki, as revised in 2024, and the study protocol was approved by the Medical Ethics Committee of the Wellbeing Services County of Southwest Finland. All participants provided written informed consent, either by a letter after receiving mailed information, or in the hospital ward after being informed of the study by the study nurse. Retrospective consent was deemed ethically acceptable due to the health status of potential study patients at the ED entry stage and the use of only leftover samples for research purposes.

### Statistical analysis

Descriptive statistics were used to analyse baseline characteristics. Categorical variables are presented as counts and percentages, while continuous variables are presented as means with standard deviations or medians with interquartile ranges, as appropriate based on distribution. Continuous variables were compared using the Mann–Whitney *U* test or Student's *t*-test, as appropriate. *χ*^2^ test was used to compare categorical variables. Correlation between long and total cTnT was assessed using Spearman’s rank correlation coefficient.

Receiver operating characteristic curve analyses were conducted to assess the area under the curve (AUC) as a measure of the ability of total cTnT and long cTnT to discriminate patients with the MI type of interest from other patients. Delong test was used to compare AUC values. We also assessed the discriminative capacity of the ratio between total cTnT and long cTnT (troponin ratio) for identifying MI. Moreover, AUC values for the combination of total and long cTnT were computed using logistic regression. We also examined the proportion of patients with MI across tertiles of total and long cTnT values. Stratified receiver operating characteristic curve analyses were conducted by sex (men and women), age (<70 or ≥70 years), estimated glomerular filtration rate (<60 or ≥60 mL/min/1.73 m²; calculated with the 2021 Chronic Kidney Disease Epidemiology Collaboration equation), time from symptom onset (<3, <12, or ≥12 h), as well as by excluding patients presenting with an ST-elevation MI. Additional analyses explored the added value of long cTnT in the clinical setting of smaller troponin elevations (total cTnT <200 ng/L).

Optimal cut-offs for total cTnT and long cTnT, maximizing sensitivity and specificity, were determined using the Youden index, and corresponding positive and negative predictive values (PPV and NPV) were calculated based on these thresholds. Additionally, specificity at a sensitivity threshold of 90% was calculated, as prioritizing sensitivity is important to minimize missed MI cases. Moreover, we assessed these diagnostic metrics for long cTnT using the 99th percentile upper reference limit of a healthy reference population (7.3 ng/L).^22^

To assess the benefit of measuring long cTnT for clinical risk classification of patients with elevated total cTnT, we used continuous net reclassification index (NRI) and decision curve analyses. The continuous NRI ranges from −2 to 2 and quantifies the number of cases whose predicted risk increases and non-cases whose predicted risk decreases with the new model compared to the reference model.^23^ Values above zero are in favour of the new model. Decision curve analyses evaluated the clinical utility of long cTnT in MI prediction, assessing the net benefit (true positive rate minus the false positive rate) in identifying MIs across a range of possible decision thresholds and comparing it to scenarios using only total cTnT data or a situation without a diagnostic biomarker.^24,25^

We assessed the added value of troponin fragmentation data in refining MI diagnosis by assessing the composite predictive capacity of using both long and total cTnT, as well as their ratio. In these analyses, predicted MI probabilities for the NRI and decision curves were calculated using nested logistic regression models with continuous variables for total cTnT, long cTnT, and their ratio, summarizing the predictive value of incorporating long cTnT measurement information into the existing total cTnT data. These probabilities were then compared to those from models using total cTnT data alone. To mitigate overfitting in the regression models, we performed repeated 10-fold random sample cross-validation, generating model predictions for each 10% data subset using models trained on the remaining 90%. Final predicted probabilities were averaged across 10 repetitions.^26^ The NRI accounts for all changes in predicted risk; however, with continuous predictors, there may be very minor shifts in predicted risks, not all of which are clinically relevant. To address this, we also computed continuous NRI by focusing only on the reclassification of patients whose predicted risk changes at least 10% (NRI_>10%_). Additionally, we performed NRI and decision curve analyses comparing long cTnT with total cTnT by using binary variables, based on the abovementioned optimal cut-off values for the MI of interest (defined by the Youden index). Bootstrapping with 1000 iterations was used to obtain the 95% confidence intervals (CIs) for the NRI. All tests were two-sided, with statistical significance assessed using a *P*-value threshold of .05 or the 95% CIs. All analyses were performed with R (version 4.2.2, R Core Team, Vienna, Austria).

## Results

The final study cohort included 1811 patients (all white Finnish population) with a total cTnT value ≥14 ng/L, of whom 1145 (63.2%) presented with chest pain or dyspnoea. Overall, 205 patients (11.3%) were diagnosed with MI, including 148 cases (8.2%) of type 1 MI and 57 cases (3.1%) of type 2 MI. Of those with type 1 MI, 40 patients had an ST-elevation MI. Patients with MI were generally younger, more often male, and had a higher prevalence of dyslipidaemia compared to patients without MI (*Table 1*). The age and sex differences were evident between patients with type 1 MI and those without MI, but not between type 2 MI and non-MI patients. Patients with MI, particularly those with type 1 MI, had chest pain more frequently and a known symptom onset time, as well as a shorter time from symptom onset (amongst those with a known onset time) compared to those without MI (*Table 1*).

**Table 1:** Characteristics of the study cohort according to type of myocardial infarction

	No MI	Any MI	*P*-value	Type 1 MI	*P*-value	Type 2 MI	*P*-value
*n*	1 606	205		148		57	
Mean age (years)	75.7 (10.8)	70.9 (12.9)	<.01	69.1 (13.5)	<.01	75.6 (9.9)	.73
Female sex	712 (44.3)	71 (34.6)	<.01	47 (31.8)	<.01	24 (42.1)	.74
Atrial fibrillation^a^	459 (28.6)	16 (7.8)	<.01	8 (5.4)	<.01	8 (14.0)	.02
Chronic kidney disease	183 (11.4)	21 (10.2)	.62	16 (10.8)	.83	5 (8.8)	.54
Coronary artery disease	463 (28.8)	66 (32.2)	.32	40 (27.0)	.64	26 (45.6)	<.01
Diabetes	525 (32.7)	58 (28.3)	.20	43 (29.1)	.37	15 (26.3)	.31
Dyslipidaemia	676 (42.1)	106 (51.7)	<.01	73 (49.3)	.09	33 (57.9)	.02
Heart failure	349 (21.7)	21 (10.2)	<.01	16 (10.8)	<.01	5 (8.8)	.02
Hypertension	1160 (72.2)	144 (70.2)	.55	100 (67.6)	.23	44 (77.2)	.41
Prior myocardial infarction	207 (12.9)	47 (22.9)	<.01	31 (20.9)	<.01	16 (28.1)	<.01
Prior stroke	188 (11.7)	15 (7.3)	.06	10 (6.8)	.07	5 (8.8)	.50
Median body mass index (kg/m^2^)	27 (24–32)	27 (25–30)	.60	27 (25–31)	.21	27 (24–30)	.32
Median estimated glomerular filtration rate (mL/min/1.73 m^2^)	70 (50–86)	79 (59–94)	<.01	81 (61–96)	<.01	75 (58–91)	.258
Admitted to hospital	1 000 (62.3)	205 (100.0)	<.01	148 (100.0)	<.01	57 (100.0)	<.01
PCI performed	20 (1.2)	124 (60.5)	<.01	108 (73.0)	<.01	16 (28.1)	<.01
Coronary artery bypass grafting planned	5 (.3)	26 (12.7)	<.01	20 (13.5)	<.01	6 (10.5)	<.01
Median highest total cardiac troponin T (ng/L)^b^	27 (19–45)	383 (122–1196)	<.01	518 (136–1713)	<.01	237 (93–461)	<.01
Classified as myocardial infarction by clinician	34 (2.1)	193 (94.1)	<.01	146 (98.6)	<.01	47 (82.5)	<.01
Known symptom onset time	835 (52.0)	189 (92.2)	<.01	138 (93.2)	<.01	51 (89.5)	<.01
Median time from symptom onset (h)^c^	11 (4–72)	6 (3–25)	<.01	5 (3–27)	<.01	8 (4–20)	<.01
Chest pain	422 (26.3)	181 (88.3)	<.01	136 (91.9)	<.01	45 (78.9)	<.01
Dyspnoea	709 (44.1)	59 (28.8)	<.01	35 (23.6)	<.01	24 (42.1)	<.01
Median 1st total cardiac troponin T (ng/L)	26 (19–42)	81 (32–220)	<.01	74 (34–232)	<.01	92 (31–190)	<.01
Median long cardiac troponin T (ng/L)	4.8 (3.0–8.4)	21.6 (8.8–74.0)	<.01	25.1 (9.5–77.5)	<.01	17.8 (6.3–58.3)	<.01
Median troponin ratio (long/total cardiac troponin T)	.17 (.10–.28)	.32 (.18–.58)	<.01	.33 (.19–.69)	<.01	.24 (.14–.45)	<.01
More than one total cardiac troponin T values taken	683 (42.5)	197 (96.1)	<.01	143 (96.6)	<.01	54 (94.7)	<.01
Median total cardiac troponin T of the 2nd sample (ng/L)^d^	31 (21–58)	255 (73–1065)	<.01	330 (82–1457)	<.01	171 (66–317)	<.01

CABG, coronary artery bypass grafting; eGFR, estimated glomerular filtration rate; BMI, body mass index; MI, myocardial infarction; PCI, percutaneous coronary intervention.

Values are presented as counts (percentages) for categorical variables, and as means (standard deviations) for normally distributed continuous variables, or medians (25th–75th percentiles) for non-normally distributed continuous variables, as specified. *P*-values are compared always to patients without any myocardial infarction.

^a^Atrial fibrillation at time of cardiac troponin T sample.

^b^During index hospitalization.

^c^In patients with known symptom onset time.

^d^In patients with 2nd total cardiac troponin T sample taken. Other than for time from symptom onset, there was no missing data for variables shown.

**Figure 1:**
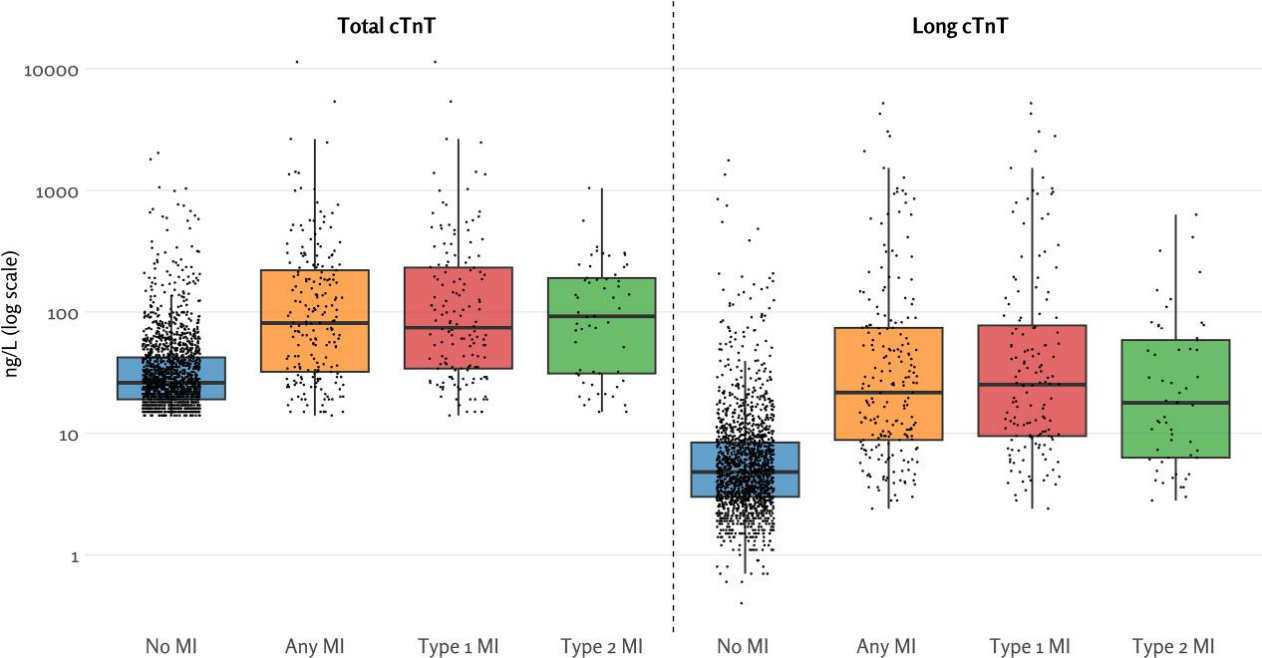
Distribution of total and long cardiac troponin T in patients with and without myocardial infarction (all *P*-values < .001 for differences between all myocardial infarction groups and no myocardial infarction)

Both long and total cTnT levels were higher in all MI categories compared to those without MI (all *P* < .001) (*Figure 1* and *Table 1*). Similarly, the troponin ratio, representing the fraction of long cTnT relative to total cTnT, was higher in all MI categories than in those without MI (median ratios 32% in any MI and 17% in those without MI; all *P* < 0. between MI types and no MI; Supplementary data online, *Figure S2*). There were no significant differences in long or total cTnT levels between patients with type 1 MI and type 2 MI (*P* = .11 and .79, respectively). However, the troponin ratio was higher in patients with type 1 MI than in those with type 2 MI (median ratios of 33% and 24%, respectively; *P* = .04; Supplementary data online, *Figure S2*). In patients without any MI, there was a weak but statistically significant correlation between long and total cTnT (*ρ* = .34, *P* < .001), whereas in patients with any MI, and particularly in those with type 1 MI, a strong correlation was observed (*ρ* = .75 and .79, respectively; both *P* < .001). Amongst patients without MI, those with higher long cTnT levels were more often male and more frequently admitted to the hospital, but had similar age, prevalence of comorbidities, proportions of chest pain and dyspnoea symptoms, as well as time from symptom onset, compared to those with lower long cTnT levels (see Supplementary data online, *Table S1*).

**Figure 2:**
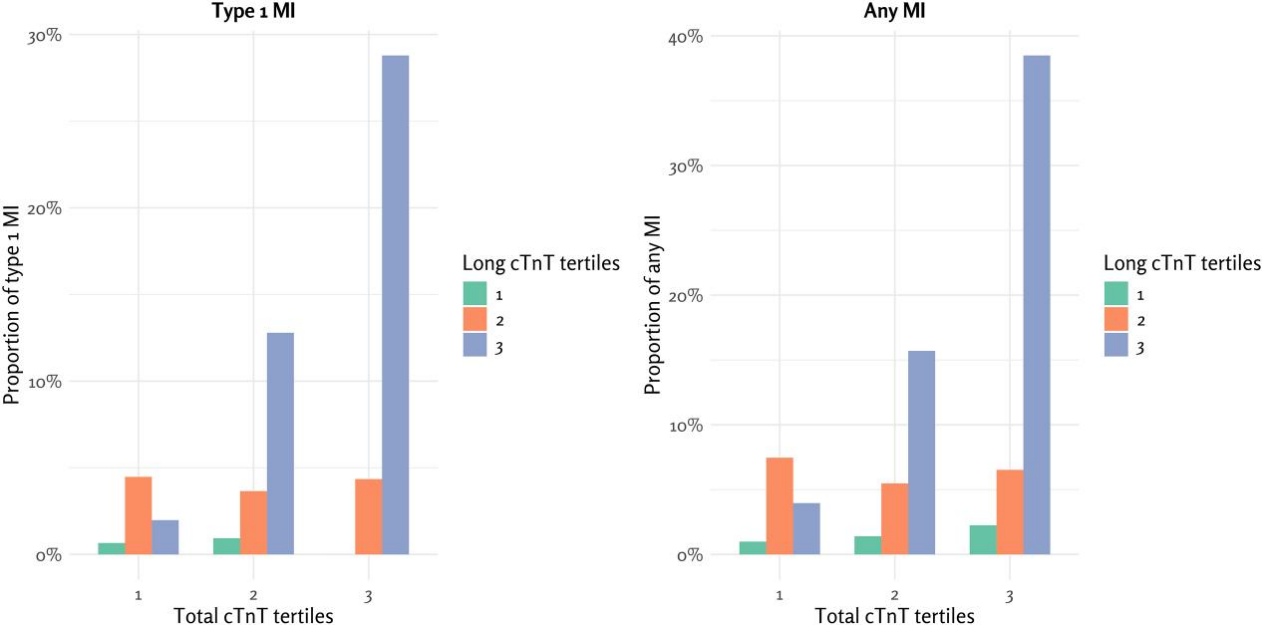
Proportion of type 1 myocardial infarction and any myocardial infarction according to the tertiles of total and long cardiac troponin T values (1 = lowest tertile, 3 = highest tertile)

When all patients were divided into tertiles based on the cTnT values, only .7% of those in the lowest long cTnT tertile had type 1 MI. In contrast, in the highest tertile of total cTnT, the proportion of type 1 MI and any MI increased substantially in higher tertiles of long cTnT (*Figure 2* and Supplementary data online, *Table S2*).

### Discriminative performance

**Figure 3:**
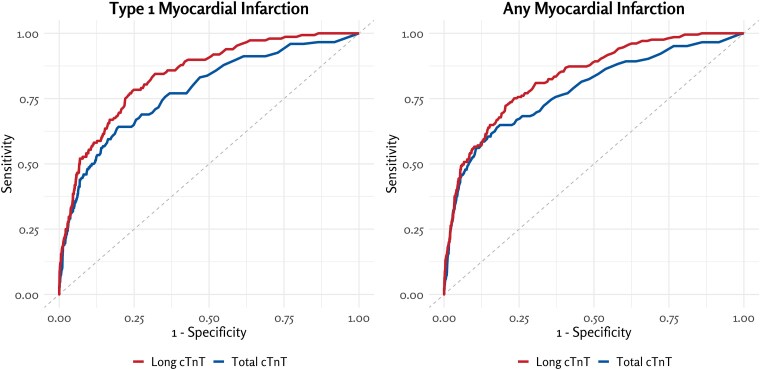
Receiver operating characteristic curves depicting the discriminative performance of total and long cardiac troponin T for differentiating type 1 myocardial infarction and any myocardial infarction from other causes of elevated troponin. Footnote: Area under the curve values for long and total cardiac troponin T: .839 vs .777 for type 1 myocardial infarction and .833 vs .782 for any myocardial infarction (both *P* < .001). These analyses cover all patients regardless of cardiac symptoms

When all patients were included in the analysis, the discriminative ability of long cTnT was superior to that of total cTnT in identifying patients with type 1 MI and any MI (AUC for type 1 MI: .839 vs .777, and for any MI: .833 vs .782, respectively, *P* < .001 for both comparisons; *Figure 3* and *Table 2*). The difference in AUC values of ROC analyses between long and total cTnT was slightly more pronounced for type 1 MI than for any MI. The specificity of long cTnT for both type 1 MI and any MI was higher (47% vs 31% and 46% vs 32%, respectively) than that of total cTnT at the threshold corresponding to 90% sensitivity. When using the 99th percentile upper reference limit cut-off from the previously published healthy reference population, the negative predictive value for type 1 MI was high at 98% (see Supplementary data online, *Table S3*). Combining total and long cTnT yielded a higher AUC than total cTnT alone, yet remaining lower than long cTnT alone (see Supplementary data online, *Table S4*). Both biomarkers performed similarly in distinguishing patients with type 2 MI from those without any MI (see Supplementary data online, *Table S5*). Troponin ratio demonstrated inferior discriminative ability compared to total cTnT for both type 1 MI and any MI (see Supplementary data online, *Table S6*). In the subcohort of patients with chest pain or dyspnoea, long cTnT outperformed total cTnT in identifying type 1 MI and any MI, aligning with findings from the overall cohort (*Table 2* and Supplementary data online, *Figure S3*).

**Table 2:** Diagnostic accuracy of troponin assays for myocardial infarction

	AUC	Optimal threshold (ng/L)	Sensitivity	Specificity	PPV	NPV	Threshold at 90% sensitivity (ng/L)	Specificity at 90% sensitivity
All patients								
Type 1 myocardial infarction (*n* = 148)								
Total cardiac troponin T	.777 (.735–.819)	51.1	64 (56–72)	80 (78–82)	22 (19–27)	96 (95–97)	20.5	31 (25–49)
Long cardiac troponin T	.839 (.807–.872)	8.8	78 (71–84)	75 (73–77)	22 (19–26)	98 (97–98)	4.6	47 (40–61)
Any myocardial infarction (*n* = 205)								
Total cardiac troponin T	.782 (.744–.819)	52.5	64 (57–70)	82 (80–84)	32 (27–36)	95 (93–96)	20.5	32 (20–46)
Long cardiac troponin T	.833 (.804–.863)	8.8	75 (69–81)	77 (75–79)	29 (25–33)	96 (95–97)	4.4	46 (39–54)
Patients with chest pain or dyspnoea								
Type 1 myocardial infarction (*n* = 142)								
Total cardiac troponin T	.756 (.711–.801)	51.1	63 (55–70)	78 (75–80)	28 (24–34)	94 (92–95)	20.5	29 (19–46)
Long cardiac troponin T	.826 (.791–.861)	8.8	78 (71–84)	73 (71–76)	29 (25–34)	96 (94–97)	4.6	45 (37–60)
Any myocardial infarction (*n* = 196)								
Total cardiac troponin T	.770 (.730–.810)	52.5	63 (56–70)	81 (78–83)	40 (35–46)	91 (89–93)	19.5	26 (19–58)
Long cardiac troponin T	.828 (.797–.859)	9.5	72 (66–78)	79 (76–82)	42 (37–47)	93 (91–95)	4.4	44 (38–58)

AUC, area under curve; MI, myocardial infarction; NPV, negative predictive value; PPV, positive predictive value. Optimal threshold based on Youden index. Sensitivity, specificity, PPV, and NPV reported as %. 95% confidence intervals in parenthesis.

All differences in area under curve values between total and long cardiac troponin T *P*-value <.001.

### Subgroup analyses

The results were largely consistent across all subgroup analyses: AUC values showed good discriminative ability for long cTnT and were higher than for total cTnT in identifying type 1 MI and any MI, regardless of sex, age, or estimated glomerular filtration rate, although the differences were not statistically significant in some subgroups with a small number of patients (see Supplementary data online, *Table S7*). The results were also similar when patients with ST-elevation MI were excluded from the analysis, as well as when the analysis was restricted to only hospitalized patients or to those with only mildly elevated total cTnT (<200 ng/L). Long cTnT displayed superior discrimination compared to total cTnT in patients presenting within 3 h (AUC values for type 1 MI .760 vs .650, *P* = .008) or within 12 h of symptom onset (AUC values for type 1 MI .807 vs .733, *P* = .001). Amongst patients with more than 12 h from symptom onset, both long and total cTnT showed good discriminative ability, and the numerical AUC difference favouring long cTnT was not statistically significant (AUC values for type 1 MI: .834 vs .804, *P* = .265; Supplementary data online, *Table S7*).

### Net reclassification index and decision curve analyses

Combining the predictive information provided by long cTnT and the troponin ratio with total cTnT improved risk classification for both type 1 MI and any MI, when compared to total cTnT alone. This was observed in the continuous NRI that captured all changes in predicted risk, as well as when considering only changes >10%, which may reflect more clinically meaningful shifts in risk stratification (*Table 3*). This finding was further supported by decision curve analyses, where the combination of total cTnT, long cTnT, and their ratio yielded higher net benefit than total cTnT alone in identifying MI patients across a range of risk threshold probabilities (see Supplementary data online, *Figure S4*).

**Table 3:** Net reclassification indices for myocardial infarction comparing predicted probabilities from combined data of total cardiac troponin T, long cardiac troponin T, and troponin ratio vs total cardiac troponin T alone

All patients		
Type 1 myocardial infarction		
NRI	0.56	(.39–.72)
NRI _>_ _10%_	0.10	(.04–.17)
Any myocardial infarction		
NRI	0.55	(.42–.70)
NRI _>_ _10%_	0.15	(.09–.22)
Patients with chest pain or dyspnoea		
Type 1 myocardial infarction		
NRI	0.67	(.51–.84)
NRI _>_ _10%_	0.12	(.04–.19)
Any myocardial infarction		
NRI	0.77	(.63–.92)
NRI _>_ _10%_	0.16	(.08–.23)

MI, myocardial infarction; NRI, net reclassification index.

NRI _>_  _10%_ accounts only for estimated risk differences >10%. Predictions averaged with 10-fold cross-validation with 10 repetitions to mitigate overfitting. NRI values range from −2 to 2, and values above zero favour the model using combined data. 95% confidence intervals in parentheses.

When comparing long cTnT vs total cTnT for classifying patients with the Youden index-based cut-off values, long cTnT demonstrated superior performance, particularly in identifying type 1 MI. This was evident in both the NRI and decision curve analyses (NRI .09, 95% CI .01–.17). For any MI, long cTnT showed a trend towards better classification, but the difference was not statistically significant (NRI .05, 95% CI −.01 to −.12; Supplementary data online, *Table S8* and Supplementary data online, *Figure S5*).

## Discussion

This prospective study assessed the diagnostic value of measuring intact and mildly truncated cTnT forms from a single admission blood sample using a novel immunoassay in ED patients with elevated high-sensitivity cTnT levels. The study has three key findings: (i) long cTnT effectively discriminates between patients with and without MI amongst those with elevated total cTnT levels; (ii) low long cTnT levels can reliably rule out MI when total cTnT levels are above the 99th percentile upper reference limit; and (iii) using long cTnT in combination with total cTnT could significantly improve diagnostic accuracy and aid clinical decision-making, particularly regarding type 1 MI (*Structured Graphical Abstract*).

The advent of high-sensitivity troponin assays has improved the detection of MI, reduced the number of missed MI cases, and enabled earlier diagnosis and treatment.^27^ However, their increased sensitivity also presents challenges, as clinicians now frequently encounter mildly elevated troponin levels in patients who do not exhibit any symptoms or signs of myocardial ischaemia. Indeed, in our study, almost 90% of patients with elevated total cTnT did not have MI. The downstream consequences of these non-MI troponin elevations, often including invasive imaging and antithrombotic therapy, can pose unnecessary risks to patients, delay the diagnostic workup of other underlying causes, and lead to overdiagnosis of MI.^2,3^ Therefore, there is a clinical need for improved accuracy in laboratory diagnostics of MI.

In the current study, amongst ED all-comers with elevated cTnT levels, long cTnT performed well in distinguishing between those with and without MI. This finding was consistent across all studied subgroups, except for symptomatic patients presenting more than 12 h after symptom onset. Importantly, the discriminatory ability of long cTnT remained high also amongst patients presenting very early after symptom onset (<3 h), as well as in those with only mildly elevated total cTnT levels. Moreover, long cTnT identified MI well also amongst patients with impaired kidney function. This finding aligns with prior evidence on the ability of long cTnT in effectively discriminating troponin elevations related to MI from those associated with end-stage kidney disease.^9,15^ The diagnostic value of long cTnT is further supported by recent data from a healthy reference population, which showed a median concentration as low as 2.0 ng/L.^22^ Overall, our findings suggest that measuring long cTnT could enhance diagnostic accuracy for MI across diverse patient populations and clinical scenarios.

The differences in discriminative performance between long and total cTnT were slightly more pronounced for type 1 MI compared to any MI, with no significant differences observed for type 2 MI. The troponin ratio, representing the proportion of long cTnT to total cTnT, was higher in patients with type 1 MI compared to those with type 2 MI, supporting the hypothesis that troponin elevations, particularly in type 1 MI, consist more of longer molecules. One pathophysiological explanation for this observation could be the typically more abrupt onset and more severe myocardial damage in type 1 MI, compared to the supply–demand imbalance that causes type 2 MI. These aspects are of clinical importance, as it is particularly type 1 MI that requires accurate diagnosis and early invasive treatment strategy, and long cTnT appears to add specificity to its diagnosis. In contrast, acute treatment of type 2 MI focuses predominantly on correcting the underlying cause of the supply–demand imbalance.^28^ While the diagnostic performance of long cTnT remained relatively good in patients with a longer time from symptom onset, the previously described time-dependent degradation of cTnT molecules after MI may explain why long cTnT appeared to lose its diagnostic advantage over total cTnT in this group.^10^ These findings are in line with the previous studies investigating degradation of cTnT as part of the complex with cTnI and troponin C and showing lower fractions of long cTnT in MI patients at later time points.^17,18,29^

Regarding clinical utility, it is important to note that all included patients presented with a suspected cardiac event and an abnormal high-sensitivity cTnT level on ED admission, but MI was diagnosed in only 11% of these cases. Given these prerequisites, the most clinically relevant finding of this study was that a low long cTnT level (<3.7 ng/L) could rule out type 1 MI with over 99% certainty in one-third of these challenging patients. This finding may have significant implications for streamlining the management of a large number of patients with non-specific total cTnT elevations in real-life clinical practice. At the other end of the spectrum, long cTnT values in the highest tertile substantially increased the likelihood of MI, even amongst those with high total cTnT levels (*Figure 2* and Supplementary data online, *Table S2*). Correspondingly, combining the predictive information of long and total cTnT demonstrated a superior net benefit than using only total cTnT in the NRI and decision curve analyses, suggesting that this combined approach could improve decision-making in the diagnosis and management of MI from a single admission blood sample.

Earlier research on troponin fragmentation has mainly utilized methods including gel filtration chromatography, Western blotting, and mass spectrometry, all of which are too complex for clinical practice.^10,16,30^ The limited analytical sensitivity of these methods presents an additional barrier to their clinical applicability. In contrast, our immunoassay approach offers a highly sensitive method for analysing cTnT fragmentation. Importantly, the principle behind our assay can be adapted for automated platforms, facilitating its integration into clinical practice to improve the laboratory diagnostics of MI. However, further research and broader validation are needed before clinical implementation.

Our study has some limitations that need to be considered. First, our study included only patients with total cTnT levels above the 99th percentile upper reference limit, and further investigation is needed in cohorts that include patients with normal admission troponin concentrations. This may also limit the direct comparison between total and long cTnT in the current study. Relatedly, some patients with MI presenting early after symptom onset may have been missed due to normal total cTnT levels at ED admission. In addition, the number of patients in type 2 MI group was limited. However, the current cohort enables the evaluation of the added value of long cTnT in the clinically relevant and challenging context of elevated high-sensitivity troponin levels amongst ED patients undergoing cardiac biomarker testing in real-world clinical practice. Relatedly, further validation studies are needed to confirm our findings in diverse patient populations, as well as to establish optimal decision thresholds for long cTnT and to determine the most effective way to integrate its predictive information into clinical practice. Further research is needed to understand long cTnT and troponin ratio profiles in acute and chronic cardiac injury, as well as in other conditions associated with elevated total cTnT, such as atrial fibrillation. Indeed, information on the mechanisms of cTnT elevation in ED patients without type 1 MI is limited. Previous reports suggest that heterogeneous pathophysiology influences the composition of troponin release, with long cTnT levels higher in Takotsubo syndrome than in end-stage kidney disease or after heavy exercise, even though smaller troponin fragments were mainly responsible for troponin elevations in all these conditions.^9,13,14^ Moreover, we did not obtain consent from a relatively large proportion of patients with early ED discharge who were contacted by mail, but most admitted patients did consent to participate in the study. This may have introduced selection bias, particularly to the control group of patients without MI. Additionally, sex-specific upper reference limits of total cTnT were not applied in the study inclusion criteria, as they are not supported by the 2023 ESC guideline for diagnosing MI, but we assume this had no meaningful impact on our results, given their consistency in sex-stratified analyses.^1^ Finally, we compared troponin values from a single admission blood sample, and guideline-recommended algorithms incorporating troponin dynamics would likely improve the accuracy of total cTnT.^1^ The dynamics of long cTnT and its diagnostic value warrant further investigation.

## Conclusions

The long cTnT assay demonstrated good diagnostic performance in ED patients with elevated total cTnT levels, with the potential to improve MI diagnosis, particularly for type 1 MI.

## Supplementary Material

ehaf975_Supplementary_Data

## Declarations

### Data Availability

The data cannot be shared publicly due to its sensitive nature.

### Pre-registered Clinical Trial Number

ClinicalTrials.gov Identifier: NCT05858112.
