# Supplementary material for: Long forms of cardiac troponin T for myocardial infarction diagnosis: the SuperTROPO study
Source: Eur Heart J. 2025 Dec 5;47(4):490–9. doi: 10.1093/eurheartj/ehaf975 (PMC12831185; doi:10.1093/eurheartj/ehaf975)
Supplement: ehaf975_Supplementary_Data [file ehaf975_supplementary_data.docx]

**Supplementary Material**

**Supplementary Table 1.** Characteristics of patients without MI according to the tertiles of long cTnT

**Supplementary Table 2.** Proportion of patients with type 1 MI or any MI in the study cohort according to tertiles of total and long cTnT.

**Supplementary Table 3.** Diagnostic Accuracy of Long cTnT Assays in Discriminating Type 1 and Any Myocardial Infarction When Using the 7.3 ng/l Cut-Off (Healthy Reference Population 99^th^ Percentile Upper Reference Limit)

**Supplementary Table 4.** Area Under the Receiver Operating Characteristic (ROC) Curve for Any Myocardial Infarction and Type 1 Myocardial Infarction Using the Combination of Total and Long cTnT.

**Supplementary Table 5.** Diagnostic Accuracy of Troponin Assays in Discriminating Type 2 Myocardial Infarction from Those Without Any Myocardial Infarction

**Supplementary Table 6.** Diagnostic Accuracy of Troponin Ratio (the ratio of long cTnT forms to total cTnT) in Discriminating Myocardial Infarction

**Supplementary Table 7.** Area Under the Curve Values for Discriminating Myocardial Infarction in Subgroups

**Supplementary Table 8.** Net reclassification indices for myocardial infarction comparing long cTnT with total cTnT using binary variables based on Youden Index-derived cut-off values.

**Supplementary Figure 1.** Flow Chart of the Patient Selection Process

**Supplementary Figure 2.** Distribution of Troponin Ratio (the ratio of long cTnT forms to total cTnT) in Patients with and without Myocardial Infarction (MI) (All p-values < 0.001 for differences between all MI groups and no MI).

**Supplementary Figure 3.** Receiver Operating Characteristic Curves Depicting the Discriminative Performance of Total and Long cTnT for Differentiating Type 1 MI and Any MI from Other Causes of Elevated Troponin in Patients with Chest Pain or Dyspnea.

**Supplementary Figure 4.** Decision Curve Analysis for Identifying Patients With Myocardial Infarction (MI) Using Total cTnT or the Combination of Total cTnT, Long cTnT, and Their Ratio.

**Supplementary Figure 5.** Decision Curve Analysis for Identifying Patients With Myocardial Infarction (MI) Comparing Total cTnT With Long cTnT Using Binary Variables Based on Youden Index-Defined Cut-Off Values.

**Supplementary Table 1.** Characteristics of patients without myocardial infarction according to the tertiles of long cTnT

|  | Long cTnT 1^st^ tertile | Long cTnT 2^nd^ tertile | Long cTnT 3^rd^ tertile | p-value |
| --- | --- | --- | --- | --- |
| Long cTnT values | <3.6 ng/L | 3.6-6.8 ng/L | >6.8 ng/L |  |
| n | 545 | 523 | 538 |  |
| Mean age (years) | 76.1 (10.9) | 75.8 (10.2) | 75.1 (11.4) | 0.364 |
| Female sex | 223 (40.9) | 226 (43.2) | 263 (48.9) | 0.025 |
| Atrial fibrillation* | 177 (32.5) | 146 (27.9) | 136 (25.3) | 0.030 |
| Coronary artery disease | 174 (31.9) | 147 (28.1) | 142 (26.4) | 0.120 |
| Diabetes | 189 (34.7) | 176 (33.7) | 160 (29.7) | 0.189 |
| Dyslipidaemia | 227 (41.7) | 232 (44.4) | 217 (40.3) | 0.401 |
| Heart failure | 113 (20.7) | 112 (21.4) | 124 (23.0) | 0.638 |
| Hypertension | 391 (71.7) | 383 (73.2) | 386 (71.7) | 0.824 |
| Prior MI | 70 (12.8) | 75 (14.3) | 62 (11.5) | 0.392 |
| Prior stroke | 59 (10.8) | 63 (12.0) | 66 (12.3) | 0.729 |
| Median eGFR (ml/min/1.73m^2^) | 67 (24-70) | 67 (24-70) | 67 (25-71) | 0.818 |
| Admitted to hospital | 319 (58.5) | 315 (60.2) | 366 (68.0) | 0.015 |
| Known symptom onset time | 263 (48.3) | 279 (53.3) | 293 (54.5) | 0.093 |
| Median time from symptom onset (h)** | 9 (3-53) | 15 (4-81) | 13 (4-75) | 0.072 |
| Chest pain | 146 (26.8) | 135 (25.8) | 141 (26.2) | 0.936 |
| Dyspnoea | 228 (41.8) | 238 (45.5) | 243 (45.2) | 0.406 |
| Median total cTnT | 20 (17-28) | 24 (18-36) | 31 (22-60) | <0.001 |
| Median troponin ratio (long/total cTnT) | 0.11 (0.08-0.16) | 0.20 (0.14-0.26) | 0.33 (0.22-0.49) | <0.001 |
| Values are presented as counts (percentages) for categorical variables, and as means (standard deviations) for normally distributed continuous variables, or medians (25th - 75th percentiles) for non-normally distributed continuous variables, as specified. Abbreviations: eGFR, estimated glomerular filtration rate; MI, myocardial infarction; * Atrial fibrillation at time of cTnT sample ** In patients with known symptom onset time. Cut-off values between the long cTnT tertiles were 3.6 ng/L and 6.8 ng/L. | | | | |

**Supplementary Table 2.** Proportion of patients with type 1 myocardial infarction (MI) or any MI (i.e. positive predictive values) and proportion of non-MI patients (i.e. negative predictive values) in the study cohort according to tertiles of total and long cTnT.

| **Type 1 MI (positive predictive value)** | | | |  |
| --- | --- | --- | --- | --- |
|  | Long cTnT 1^st^ tertile | Long cTnT 2^nd^ tertile | Long cTnT 3^rd^ tertile | All |
| Total cTnT 1^st^ tertile | 0.7% | 4.5% | 2.0% | 2.2% |
| Total cTnT 2^nd^ tertile | 0.9% | 3.7% | 12.8% | 5.3% |
| Total cTnT 3^rd^ tertile | 0.0% | 4.3% | 28.8% | 17.1% |
| All | 0.7% | 4.1% | 19.7% |  |
| **Any MI (positive predictive value)** | | | |  |
|  | Long cTnT 1^st^ tertile | Long cTnT 2^nd^ tertile | Long cTnT 3^rd^ tertile | All |
| Total cTnT 1^st^ tertile | 1.0% | 7.5% | 4.0% | 3.6% |
| Total cTnT 2^nd^ tertile | 1.4% | 5.5% | 15.7% | 7.0% |
| Total cTnT 3^rd^ tertile | 2.2% | 6.5% | 38.5% | 23.4% |
| All | 1.3% | 6.5% | 26.2% |  |
| **No type 1 MI (negative predictive value)** | | | | |
|  | Long cTnT 1^st^ tertile | Long cTnT 2^nd^ tertile | Long cTnT 3^rd^ tertile | All |
| Total cTnT 1^st^ tertile | 99.3% | 95.5% | 98.0% | 97.8% |
| Total cTnT 2^nd^ tertile | 99.1% | 96.3% | 87.2% | 94.7% |
| Total cTnT 3^rd^ tertile | 100% | 95.7% | 71.2% | 82.9% |
| All | 99.3% | 95.9% | 80.3% |  |
| **No any MI (negative predictive value)** | | | | |
|  | Long cTnT 1^st^ tertile | Long cTnT 2^nd^ tertile | Long cTnT 3^rd^ tertile |  |
| Total cTnT 1^st^ tertile | 99.0% | 92.5% | 96.0% | 96.4% |
| Total cTnT 2^nd^ tertile | 98.6% | 94.5% | 84.3% | 93.0% |
| Total cTnT 3^rd^ tertile | 97.8% | 93.5% | 61.5% | 76.6% |
| All | 98.7% | 93.5% | 73.8% |  |
| The values represent the proportion of patients in each category with MI of interest. Cut-off values for the total cTnT tertiles were 22.0 ng/L and 38.0 ng/L, and for the long cTnT tertiles 3.7 ng/L and 7.8 ng/L. All differences within tertiles p<0.01. | | | | |

**Supplementary Table 3.** Diagnostic accuracy of long cTnT assays in discriminating type 1 and any myocardial infarction when using the 7.3 ng/l cut-off (Healthy reference population 99^th^ percentile upper reference limit)

|  |  | Sensitivity | Specificity | PPV | | NPV | | |
| --- | --- | --- | --- | --- | --- | --- | --- | --- |
| **All patients** | | | | |  | |  | |
| Type 1 MI |  | 84 (77-89) | 69 (66-71) | 19 (16-22) | | 98 (97-99) | | |
| Any MI |  | 80 (74-85) | 70 (68-72) | 25 (22-29) | | 97 (95-97) | | |
| Abbreviations: MI, myocardial infarction; NPV, negative predictive value; PPV, positive predictive value. Sensitivity, specificity, PPV and NPV reported as %. 95% confidence intervals in parenthesis. | | | | | | | |  |

**Supplementary Table 4.** Area under the receiver operating characteristic (ROC) curve for any myocardial infarction and type 1 myocardial infarction using the combination of total and long cTnT.

|  | **AUC** |
| --- | --- |
| **All patients** | |
| **Type 1 MI** |  |
| Total cTnT | 0.777 (0.735-0.819) |
| Total cTnT + Long cTnT | 0.802 (0.763-0.841) |
| **Any MI** |  |
| Total cTnT | 0.782 (0.744-0.819) |
| Total cTnT + Long cTnT | 0.801 (0.766-0.836) |
| **Patients with chest pain or dyspnea** | |
| **Type 1 MI** |  |
| Total cTnT | 0.756 (0.711-0.801) |
| Total cTnT + Long cTnT | 0.781 (0.739-0.823) |
| **Any MI** |  |
| Total cTnT | 0.770 (0.730-0.810) |
| Total cTnT + Long cTnT | 0.788 (0.750-0.826) |
| All differences in AUC values between total and long cTnT p-value <0.001. Abbreviations: AUC, area under curve; MI, myocardial infarction. 95% confidence intervals in parenthesis. | |

**Supplementary Table 5.** Diagnostic accuracy of troponin assays in discriminating type 2 myocardial infarction from those without any myocardial infarction

|  | AUC | | Optimal threshold (ng/L) | | Sensitivity | Specificity | | PPV | NPV | Threshold at 90% sensitivity (ng/L) | Specificity at 90% sensitivity |
| --- | --- | --- | --- | --- | --- | --- | --- | --- | --- | --- | --- |
| **All patients** | | | | | | | |  | |  |  |
| **Type 2 MI** (n= 57) | |  |  |  | | |  |  | |  |  |
| Total cTnT | 0.770 (0.694-0.845) | | 69.5 | | 63 (50-75) | 90 (88-91) | | 18 (13-24) | 99 (98-99) | 19.5 | 28 (8-47) |
| Long cTnT | 0.794 (0.733-0.854) | | 9.8 | | 65 (52-76) | 81 (79-83) | | 11 (8-14) | 99 (98-99) | 4.0 | 40 (25-51) |
| **Patients with chest pain or dyspnea** | | | | | | | | | |  |  |
| **Type 2 MI** (n= 54) | |  |  |  | | |  |  | |  |  |
| Total cTnT | 0.769 (0.690-0.847) | | 69.5 | | 65 (52-76) | 89 (86-90) | | 24 (18-32) | 98 (96-99) | 19.5 | 26 (7-48) |
| Long cTnT | 0.793 (0.730-0.855) | | 9.8 | | 67 (53-78) | 81 (78-83) | | 16 (12-22) | 98 (96-99) | 4.2 | 41 (23-51) |
| AUC differences between total and long cTnT: p = 0.527 (all patients), p = 0.500 (symptomatic patients). Abbreviations: AUC, area under curve; MI, myocardial infarction; NPV, negative predictive value; PPV, positive predictive value. Optimal threshold based on Youden Index. Sensitivity, specificity, PPV and NPV reported as %. 95% confidence intervals in parenthesis. | | | | | | | | | | | |

**Supplementary Table 6.** Diagnostic accuracy of troponin ratio (the ratio of long cTnT forms to total cTnT) in discriminating myocardial infarction

|  | AUC | | Optimal threshold | | | | Sensitivity | | Specificity | | | PPV | NPV | Threshold at 90% sensitivity | Specificity at 90% sensitivity |
| --- | --- | --- | --- | --- | --- | --- | --- | --- | --- | --- | --- | --- | --- | --- | --- |
| **All patients** | | | | | | | | | | | |  | |  |  |
| **Type 1 MI** (n= 148) | |  |  | |  | | | | |  | |  | |  |  |
| Total cTnT | 0.777 (0.735-0.819) | | 51.1 ng/L | | | | 64 (56-72) | | 80 (78-82) | | | 22 (19-27) | 96 (95-97) | 20.5 ng/L | 31 (25-49) |
| Troponin ratio | 0.711 (0.664-0.757) | | 0.26 | | | | 65 (57-72) | | 71 (69-73) | | | 17 (14-20) | 96 (95-97) | 0.09 | 19 (6-33) |
| **Any MI** (n= 205) | |  |  |  | |  | |  | | |  |  |  |  |  |
| Total cTnT | 0.782 (0.744-0.819) | | 52.5 ng/L | | | | 64 (57-70) | | 82 (80-84) | | | 32 (27-36) | 95 (93-96) | 20.5 ng/L | 32 (20-46) |
| Troponin ratio | 0.687 (0.645-0.729) | | 0.26 | | | | 61 (54-67) | | 72 (70-74) | | | 22 (19-25) | 94 (92-95) | 0.08 | 14 (4-22) |
| **Patients with chest pain or dyspnoea** | | | | | | | | | | | | | |  |  |
| **Type 1 MI** (n= 142) | |  |  | |  | | | | |  | |  | |  |  |
| Total cTnT | 0.756 (0.711-0.801) | | 51.1 ng/L | | | | 63 (55-70) | | 78 (75-80) | | | 28 (24-34) | 94 (92-95) | 20.5 | 29 (19-46) |
| Troponin ratio | 0.708 (0.660-0.756) | | 0.26 | | | | 64 (56-72) | | 70 (67-73) | | | 23 (19-28) | 93 (91-95) | 0.09 | 20 (11-33) |
| **Any MI** (n= 196) | |  |  | | | |  | |  | | |  |  |  |  |
| Total cTnT | 0.770 (0.730-0.810) | | 52.5 ng/L | | | | 63 (56-70) | | 81 (78-83) | | | 40 (35-46) | 91 (89-93) | 19.5 | 26 (19-58) |
| Troponin ratio | 0.685 (0.641-0.729) | | 0.26 | | | | 60 (53-67) | | 71 (68-74) | | | 30 (26-35) | 90 (87-92) | 0.08 | 14 (5-22) |
| Abbreviations: AUC, area under curve; MI, myocardial infarction; NPV, negative predictive value; PPV, positive predictive value. Optimal threshold based on Youden Index. Sensitivity, specificity, PPV and NPV reported as %. 95% confidence intervals in parenthesis. | | | | | | | | | | | | | | | |

**Supplementary Table 7.** Area under the curve values for discriminating myocardial infarction in subgroups

|  | **AUC** | **p-value** | **AUC** | **p-value** |
| --- | --- | --- | --- | --- |
|  | **Men** | | **Women** | |
| **Type 1 MI** |  | n=101 |  | n=47 |
| Total cTnT | 0.782 (0.731-0.833) | Reference | 0.766 (0.688-0.844) | Reference |
| Long cTnT | 0.850 (0.812-0.888) | <0.001 | 0.823 (0.762-0.883) | 0.100 |
| **Any MI** | n=134 | | n=71 | |
| Total cTnT | 0.779 (0.733-0.825) | Reference | 0.786 (0.721-0.851) | Reference |
| Long cTnT | 0.841 (0.805-0.877) | 0.001 | 0.825 (0.774-0.875) | 0.191 |
|  | **Age<70 years** | | **Age ≥70 years** | |
| **Type 1 MI** |  | n=69 |  | n=79 |
| Total cTnT | 0.689 (0.539-0.840) | Reference | 0.775 (0.715-0.834) | Reference |
| Long cTnT | 0.794 (0.699-0.889) | 0.053 | 0.857 (0.817-0.897) | <0.001 |
| **Any MI** |  | n=84 |  | n=121 |
| Total cTnT | 0.780 (0.723-0.837) | Reference | 0.784 (0.734-0.833) | Reference |
| Long cTnT | 0.823 (0.774-0.872) | 0.064 | 0.834 (0.796-0.872) | 0.021 |
|  | **eGFR <60 mL/min/1.73 m²** | | **eGFR≥60 mL/min/1.73 m²** | |
| **Type 1 MI** |  | n=36 |  | n=112 |
| Total cTnT | 0.776 (0.688-0.865) | Reference | 0.795 (0.747-0.843) | Reference |
| Long cTnT | 0.866 (0.814-0.919) | 0.019 | 0.830 (0.791-0.896) | 0.053 |
| **Any MI** |  | n=52 |  | n=153 |
| Total cTnT | 0.754 (0.672-0.836) | Reference | 0.808 (0.767-0.849) | Reference |
| Long cTnT | 0.858 (0.811-0.905) | 0.004 | 0.825 (0.789-0.861) | 0.338 |
|  | **Time from symptoms <12h** | | **Time from symptoms ≥12h** | |
| **Type 1 MI** |  | n=88 |  | n=46 |
| Total cTnT | 0.733 (0.673-0.794) | Reference | 0.804 (0.732-0.876) | Reference |
| Long cTnT | 0.807 (0.758-0.857) | 0.001 | 0.834 (0.773-0.895) | 0.265 |
| **Any MI** |  | n=117 |  | n=66 |
| Total cTnT | 0.754 (0.699-0.808) | Reference | 0.810 (0.747-0.873) | Reference |
| Long cTnT | 0.814 (0.769-0.860) | 0.010 | 0.839 (0.787-0.892) | 0.282 |
|  | **Only patients admitted to hospital** | |  | |
| **Type 1 MI** |  | n=148 |  |  |
| Total cTnT | 0.736 (0.690-0.782) | Reference |  |  |
| Long cTnT | 0.818 (0.784-0.853) | <0.001 |  |  |
| **Any MI** |  | n=205 |  |  |
| Total cTnT | 0.745 (0.704-0.786) | Reference |  |  |
| Long cTnT | 0.816 (0.785-0.848) | <0.001 |  |  |
|  | **Time from symptoms <3h** | |  |  |
| **Type 1 MI** |  | n=38 |  |  |
| Total cTnT | 0.650 (0.546-0.753) | Reference |  |  |
| Long cTnT | 0.760 (0.677-0.844) | 0.008 |  |  |
| **Any MI** |  | n=46 |  |  |
| Total cTnT | 0.683 (0.588-0.777) | Reference |  |  |
| Long cTnT | 0.787 (0.711-0.863) | 0.007 |  |  |
| **Patients with total cTnT<200 ng/L** | | |  | |
| **Type 1 MI** |  | n=106 |  |  |
| Total cTnT | 0.717 (0.665-0.770) | Reference |  |  |
| Long cTnT | 0.804 (0.763-0.844) | <0.001 |  |  |
| **Any MI** |  | n=149 |  |  |
| Total cTnT | 0.723 (0.676-0.796) | Reference |  |  |
| Long cTnT | 0.796 (0.760-0.832) | <0.001 |  |  |
|  | **Patients without STEMI** | |  |  |
| **Type 1 MI** |  | n=108 |  |  |
| Total cTnT | 0.775 (0.725-0.824) | Reference |  |  |
| Long cTnT | 0.830 (0.792-0.867) | 0.003 |  |  |
| **Any MI** |  | n=165 |  |  |
| Total cTnT | 0.780 (0.738-0.821) | Reference |  |  |
| Long cTnT | 0.824 (0.791-0.857) | 0.013 |  |  |
| Abbreviations: AUC, area under curve; eGFR, estimated glomerular filtration rate; MI, myocardial infarction; STEMI, ST-elevation myocardial infraction. The count (n) represent the number of patients with myocardial infarction in each category. The analyses stratified based on time from symptom onset included only patients with chest pain or dyspnea and excluded those with unknown symptom onset times (Total number of patients in these groups was 144 (<3 hours), 410 (<12 hours), and 387 (≥12 hours). 95% confidence intervals in parenthesis. | | | | |

**Supplementary Table 8.** Net reclassification indices for myocardial infarction comparing long cTnT with total cTnT using binary variables based on Youden Index-derived cut-off values.

| **All patients** | | |
| --- | --- | --- |
| **Type 1 MI** |  |  |
| NRI | 0.09 | (0.01-0.17) |
| **Any MI** |  | |
| NRI | 0.05 | (-0.01-0.12) |
| **Patients with chest pain or dyspnoea** | | |
| **Type 1 MI** |  |  |
| NRI | 0.11 | (0.03-0.19) |
| **Any MI** |  |  |
| NRI | 0.06 | (-0.01-0.13) |
| Abbreviations: MI, myocardial infarction; NRI, net reclassification index. 95% confidence intervals in parenthesis. Long and total cTnT were used as binary variables based on Youden Index-derived optimal cut-off values for the outcome of interest. NRI values range from -2 to 2 and values above zero are in favor of the long cTnT. | | |

**Supplementary Figure 1.** Flow chart of the patient selection process


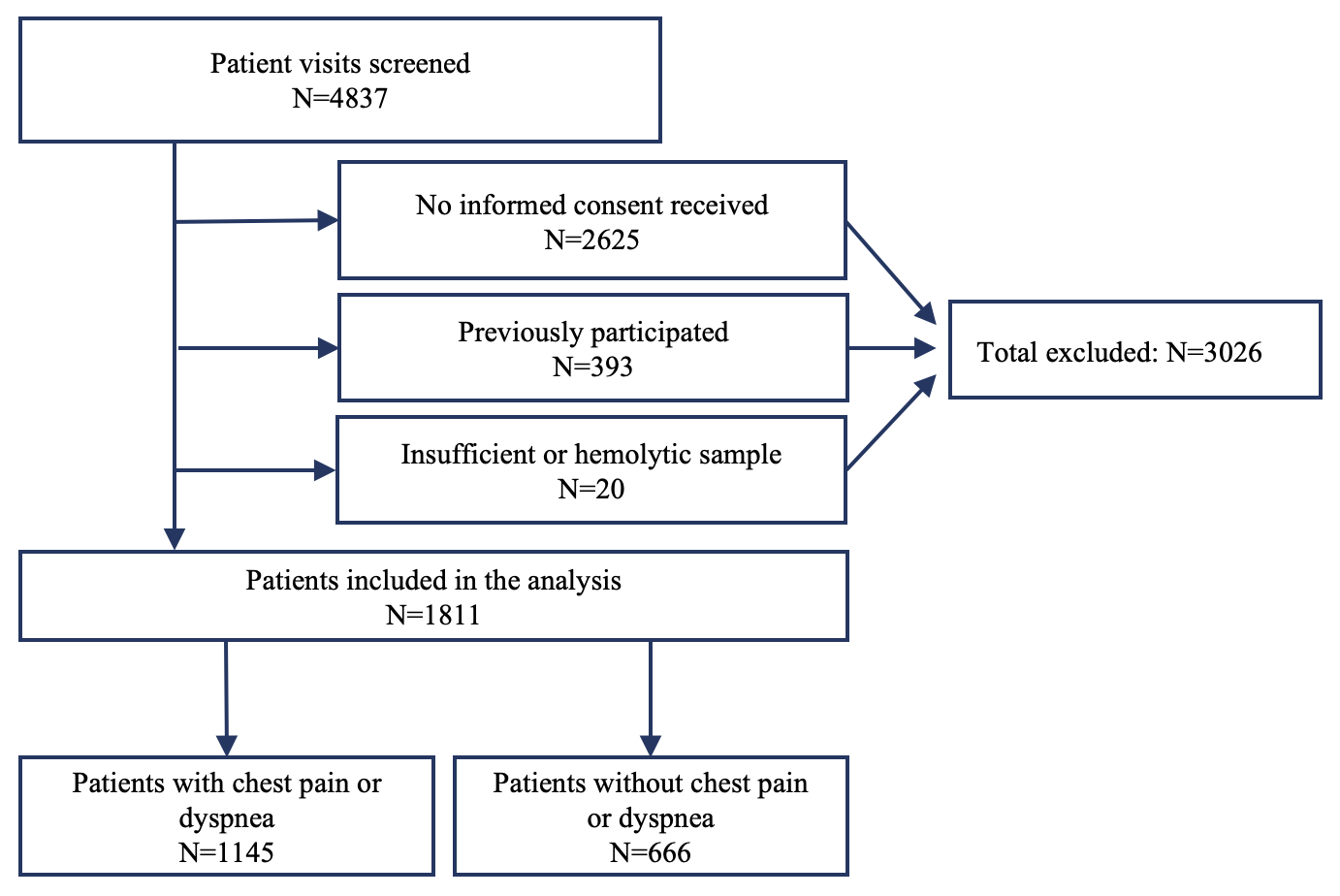


**Supplementary Figure 2.** Distribution of troponin ratio (the ratio of long cTnT forms to total cTnT) in patients with and without myocardial infarction (MI) (All p-values < 0.01 for differences between all MI groups and no MI). In some patients the long cTnT concentrations were higher than the total cTnT values. This is likely due to differences in the calibration of the assays.

**Supplementary Figure 3.** Receiver operating characteristic curves depicting the discriminative performance of total and long cTnT for differentiating type 1 myocardial infarction (MI) and any MI from other causes of elevated troponin in patients with chest pain or dyspnoea.

Footnote: Area under the curve values for total and long cTnT: 0.756 vs. 0.826 for type 1 MI and 0.770 vs. 0.828 for any MI, respectively (both p < 0.001).

**Supplementary Figure 4.** Decision curve analysis for identifying patients with myocardial infarction (MI) using total cTnT or the combination of total cTnT, long cTnT, and their ratio.

Footnote: The x-axis represents the risk threshold preference for diagnosing MI and the y-axis depicts the net benefit (calculated as the true positive rate minus the false positive rate).

**Supplementary Figure 5.** Decision curve analysis for identifying patients with myocardial infarction (MI) comparing total cTnT with long cTnT using binary variables based on Youden index-defined cut-off values.

Footnote: The x-axis represents the risk threshold preference for diagnosing MI and the y-axis depicts the net benefit (calculated as the true positive rate minus the false positive rate).
